# History of trauma is a critical treatment target for individuals at clinical high-risk for psychosis

**DOI:** 10.3389/fpsyt.2022.1102464

**Published:** 2023-01-04

**Authors:** Vanessa C. Zarubin, Tina Gupta, Vijay A. Mittal

**Affiliations:** ^1^Department of Psychology, Northwestern University, Evanston, IL, United States; ^2^Institute for Innovations in Developmental Sciences (DevSci), Northwestern University, Chicago, IL, United States; ^3^Department of Psychiatry, University of Pittsburgh, Pittsburgh, PA, United States; ^4^Department of Psychiatry, Northwestern University, Chicago, IL, United States; ^5^Medical Social Sciences, Northwestern University, Chicago, IL, United States; ^6^Institute for Policy Research (IPR), Northwestern University, Chicago, IL, United States

**Keywords:** clinical high-risk (CHR) for psychosis, trauma, treatment development, childhood trauma and adversity, psychosis

## Abstract

People meeting criteria for a clinical high-risk (CHR) for psychosis syndrome frequently represent a heterogeneous, help-seeking, and dynamic population. Among the numerous symptoms and risk factors for psychosis, exposure to trauma stands out as both highly prevalent and poorly understood. Indeed, while up to 80% of individuals meeting criteria for a CHR syndrome report trauma histories, there is currently limited research dedicated to this specific area. This is particularly problematic as trauma is tied to risk for conversion, leads to a range of clinical issues, and contributes to disability and poor quality of life. Fortunately, recent research in the general population has led to a significant evolution in the way trauma is assessed and understood, and further, some studies have indicated that targeted trauma interventions in formal psychotic disorders are highly effective. However, direct adoption is challenging as the CHR syndrome holds a number of unique concerns (e.g., clinical heterogeneity, developmental trauma), and characteristically, involves a developing pediatric or young adult population that also comes with specific considerations (e.g., living with caregivers, transitionary period in roles). In this “perspective” we frame the issues around understanding trauma in CHR individuals, discuss viable treatments and unique considerations, and provide suggestions for future steps in developing and incorporating trauma-focused interventions in this population.

## 1. Introduction

Evidence suggests that individuals diagnosed with a psychotic disorder such as schizophrenia are significantly more likely to have had exposure to trauma [i.e., psychological, physical, emotional, or sexual abuse and emotional neglect; (1, 2)]. In schizophrenia, trauma is often not a focus given historical concerns that trauma-focused interventions (treatments directed at addressing the trauma and sequelae) would exacerbate psychotic symptoms (3). Trauma exposure is just beginning to be understood among those considered at clinical high-risk (CHR) for psychosis, who are at imminent risk for developing psychosis (4). It is of particular importance for this population as trauma exposure increases the odds of developing a psychotic disorder by nearly a factor of 3 (5).

Trauma exposure is highly prevalent among individuals with a CHR syndrome, with estimates as high as 86.5% having at least one exposure (6). Further, many individuals presenting with CHR symptoms also exhibit trauma-related symptoms and may have a post-traumatic stress disorder (PTSD) diagnosis. Given evidence to suggest trauma exposure predicts conversion to a psychotic disorder (5, 7, 8), studying it in those with a CHR syndrome can inform early intervention efforts. Indeed, the number of types of trauma experienced is one of only eight items included in the NAPLS Risk Calculator, used to estimate probabilistic risk of an individual converting to full-threshold psychosis (7). Additionally, trauma-related exposure is linked to higher severity of attenuated psychotic symptoms (9). This provides additional evidence of the importance of examining the nature of trauma exposure during this vulnerable, developmental window.

Importantly, there is considerable research into early intervention treatment broadly in those with a CHR syndrome. Interventions showing promise include cognitive behavioral therapy (CBT), cognitive remediation, app-based interventions, medications such as antipsychotics or selective serotonin reuptake inhibitors, fish oil, cannabidiol, and social skills training (10–18). Many treatments target attenuated psychosis symptoms indirectly by addressing other areas of concern (e.g., depression and anxiety) or by strengthening coping strategies (e.g., social skills training). Despite the established role of trauma in increasing vulnerability for developing a psychotic disorder, there has yet to be research examining trauma-focused treatment in those with a CHR syndrome. Application and adaptation of evidence-supported treatments for trauma-related symptoms to the CHR population is urgently needed.

In the present perspective, the goal is to discuss trauma exposure, with an emphasis on our understanding of trauma exposure in those with a CHR syndrome. Furthermore, we discuss unique considerations and intervention strategies, drawing from the schizophrenia and trauma literature.

## 2. Trauma exposure and treatments in schizophrenia

Psychosis is associated with considerable disease burden due to the early onset and chronic course of symptoms (19). The interaction between trauma exposure and psychosis can be devasting and further contribute to disability (5, 7, 8). The well-established stress-vulnerability model emphasizes the ways in which biological changes related to stress exposure interact with underlying predispositional vulnerability (e.g., genetic risk) to result in the manifestation of mental illness (20, 21). Specifically, chronic stress elicits persistently elevated levels of cortisol, leading to an imbalance in pro- and anti-inflammatory cytokines (20). This dysregulation results in neurodegenerative changes in the hippocampus, which is linked with numerous psychiatric disorders (20). The extension of this model to the vulnerability-stress-inflammation model of schizophrenia highlights how the many sequelae of chronic stress (e.g., increased microglia activation, loss of central nervous system volume, alterations in glutamatergic neurotransmission) may be pathophysiological mechanisms as well as potential treatment targets (22).

Additionally, the traumagenic neurodevelopmental model of psychosis focuses on the causal role childhood trauma may have in the development of psychotic disorders, with considerable support (8, 23). This models posits that the heightened sensitivity to stress seen in individuals diagnosed with a psychotic disorder is related to neurodevelopmental changes in the brain following trauma exposure (8). Biological changes in the brain observed in psychosis and early childhood adversity are strikingly similar, including overactivity of the hypothalamic-adrenal-pituitary (HPA) axis, abnormalities in dopamine, serotonin, and norepinephrine, hippocampal changes, cerebral atrophy, ventricular enlargements, and altered cerebral asymmetry (8, 24). The phenomenology of PTSD symptoms and some psychotic-like symptoms also overlap substantially (8). This suggests responses to trauma exposure and the development of psychotic disorders may share mechanisms, which could inform treatment.

As noted, many practitioners were concerned about conducting trauma-related treatments with individuals experiencing psychosis (3). Recent research has demonstrated that both trauma-informed approaches (treatments considering but not directly addressing trauma and its sequelae, e.g., Cognitive Behavioral Therapy for Psychosis; CBTp) and trauma-focused treatments (e.g., Eye Movement Desensitization and Reprocessing; EMDR, Prolonged Exposure; PE) are effective in psychosis populations, without exacerbation or destabilization of psychotic symptoms. One exception is PE which demonstrated temporary symptom exacerbation, which has also been shown in non-psychotic populations (25). To date, the current literature reflects strong support for the use of EMDR and PE, with more mixed results for cognitive restructuring [CR; (23)]. PTSD treatments support a generalization effect where, following the treatment of trauma-related symptoms, other symptoms (e.g., depression, anxiety) also improve. The more limited literature on trauma-focused treatment in individuals with both PTSD and psychotic symptoms also supports generalization; following trauma treatment, the frequency and/or intensity of psychotic symptoms, particularly those related to suspiciousness, paranoia, or trauma-dependent hallucinations, decreased (9, 24–26). A planned feasibility study on EMDR for psychosis (EMDRp) shows promise as an add-on to treatment (9), but results have not yet been published.

Little research, however, has examined incorporating trauma-treatment protocols with care for psychosis instead of adding them adjunctively. One notable exception is Trauma-Integrated Cognitive Behavioral Therapy for Psychosis [TI-CBTp; (27)]. This protocol was implemented in a Coordinated Specialty Care (CSC) model clinic treating individuals experiencing sub-threshold or first-episode psychotic symptoms. Critically, the model triages the focus of treatment taking into consideration the presence, relative severity, and stability of both psychotic and trauma-related symptoms, facilitating the appropriate timing of treatment for each (27). Early support indicates that psychosis symptoms were not exacerbated by trauma-integrated treatment, and that engagement resulted in reduced symptoms (27).

Despite the promising results of the TI-CPTp protocol, it does not explicitly consider the differences between those with a CHR syndrome and those with a recent onset of a psychotic episode, which potentially include younger ages, different contexts (e.g., work vs. school), and different treatment goals. These differences, discussed later in the perspective, emphasize the importance of tailoring treatment to the CHR population.

## 3. Trauma treatments in those with a CHR syndrome

Research has brought attention to the possibilities of and need for trauma-focused treatments in the CHR period for years (6, 24); however, only limited research has been published examining the possibility of implementing these treatments. The study discussed above included three youth at CHR in the protocol (27); however, there was no discussion of modification to consider factors unique to the CHR population.

General early intervention treatments for the CHR population often includes treatment for comorbid diagnoses or provides instruction in the development of coping skills aimed to reduce stress or improve functioning (10, 14–16, 18). This focus on reduction of stress experienced is critical, given that individuals at CHR have higher stress reactivity than both non-psychiatric controls and psychotic patients (28–32).

While targeting central processes and symptoms (e.g., positive symptoms, stress) may be useful in the context of trauma treatment, there is still a need to develop trauma-focused therapies that can be particularly effective for trauma specific/related symptoms (e.g., emotion dysregulation, stress sensitivity) if these are the main presenting concern for an individual with CHR symptoms. Existing evidence-supported treatments designed to target the sequelae of trauma exposure such as Trauma-Focused Cognitive Behavioral Therapy (TF-CBT), PE, and EMDR, are known to be effective in general trauma treatment and in individuals with psychosis, as mentioned (9, 24–26, 33). Adaptations and implementation of these treatments in early intervention for individuals at CHR have the potential to be highly effective in reducing trauma-related symptomatology and, potentially, the risk for developing a psychotic disorder. Additionally, application of third-wave approaches in the treatment of symptoms resulting from trauma or invalidation is an understudied area. Third-wave approaches such as Dialectical Behavior Therapy (DBT) and Acceptance and Commitment Therapy [ACT; (34)] may be useful and perhaps adapted to CHR populations. Given DBT, for example, targets emotion dysregulation (35), and emotion dysregulation is common in those with a CHR syndrome (6) and is linked to trauma exposure broadly (8), there may be utility in this approach. DBT and ACT have been shown to be effective for emotion dysregulation, binge eating, depression, anxiety, and hallucinations (36, 37) which provides support for their possible efficacy in addressing auxiliary symptoms resulting from trauma exposure. While the full protocol of these approaches may not be as effective as trauma-informed therapies, there could be benefits in adapting and applying specific skills (e.g., increasing distress tolerance).

One critical factor in trauma-focused treatment development is that one single therapy may not be adequate for those with a CHR syndrome, given the complex and varied stressors they face. This provides support to taking integrative approaches in developing and applying interventions for trauma in those with a CHR syndrome. Additionally, systems-level interventions targeting problems such as bullying in schools, poverty, crime exposure, and systemic discrimination are direly needed. These high-level stressors increase risk for psychotic disorders at the population level (38), with the greatest impact on marginalized groups. As additional research is being published, evidence for this link is strengthening. Without adequate attention and resources devoted to addressing systemic problems with systemic solutions, additional strain will be placed on the already overburdened mental health care system as it attempts to compensate at the individual level.

## 4. Unique considerations in trauma treatments in those with a CHR syndrome

While there is evidence of effective trauma treatments in schizophrenia such as TI-CBTp and possibilities to adapt current interventions for CHR groups, there are important considerations specific to the CHR syndrome. For example, individuals meeting criteria for the CHR syndrome are often younger, meaning some treatment considerations relevant for adults are not generally applicable (e.g., job skills, independent living), and other considerations must be included in treatment planning. Individuals at CHR often still live with caregivers, may have increased or different internet interactions, and are frequently in school contexts where bullying and other stressors may be present. This could create delicate situations requiring special care if, for example, the individual is still exposed to the situation that led to developmental trauma with limited control over how and whether they engage. Additionally, individuals at CHR are in a transitionary period in which societal roles and symptoms may shift dramatically as the individual navigates pre-adolescence, adolescence, or young adulthood, and treatment should be tailored with this context in mind.

Another important consideration is clinical heterogeneity and comorbidity for CHR groups. Given the heterogeneity in symptoms in this group (39–43), the need to assess main presenting concerns is critical. When assessing, it can be valuable to identify symptoms outside of positive symptoms. Trauma exposure can result in a range of clinically relevant experiences, including re-experiencing the event(s) (e.g., flashbacks, nightmares), avoidance of internal and/or external reminders, persistent negative changes in thinking and mood (e.g., thinking no one can be trusted, inability to feel positive emotions), and changes in physical and emotional reactions [e.g., hypervigilance, difficulty concentrating, irritability; (21)]. Trauma exposure can also result in increased stress sensitivity and increased risk for internalizing (e.g., depression, anxiety, anhedonia) and externalizing problems [e.g., impulsivity, aggression, substance abuse; (41)]. The increased risk for psychopathology can result in more complex clinical presentations, increasing the need for adequate assessment and tailored clinical care.

Individuals at CHR for psychosis often exhibit characteristics of a general distress and impairment which is associated with broad, pluripotent risk for developing psychopathology that turns into different endophenotypes following the emergence of symptoms with more diagnostic specificity (44–46). This may present with broad psychopathology consisting of multiple symptoms appearing together, such as the internalizing and externalizing symptoms discussed above. Notably, the distress syndrome itself is often clinically relevant and can be effectively treated by targeting commonly occurring difficulties such as emotion dysregulation, which are more likely to occur following trauma exposure. Not only does reduced overall stress and improved coping reduce the likelihood of conversion to a psychotic disorder, treatment of these general targets can result in the reduction or elimination of attenuated symptoms (10), and incorporating trauma-focused treatment may result in greater improvements.

Many individuals meeting criteria for a CHR syndrome, especially those with multiple trauma exposures (e.g., repeated physical abuse or experiencing both physical and emotional abuse) and the associated higher risk for developing psychotic disorders, have experienced trauma in developmental contexts. Childhood trauma, or developmental trauma, is often defined as trauma exposures occurring prior to the age of 17, when trauma may influence developmental processes (5). As such, reactions may be different or more extensive than those of individuals exposed to trauma as adults. For example, the proposed criteria for developmental trauma disorder (DTD) include emotion dysregulation, somatic dysregulation, impaired access to emotion or somatic feelings, and impaired emotion or somatic verbal mediation/expression (47). Affected individuals also exhibit attention bias toward or away from threat, impaired self-protection, maladaptive self-soothing, non-suicidal self-injury, or impaired ability to initiate or sustain goal-directed behavior. Finally, criteria include self-loathing (including seeing the self as irreparably damaged or defective), attachment insecurity and disorganization, betrayal-based relational schemas, reactive verbal or physical aggression, impaired psychological boundaries, or impaired interpersonal empathy (47). Many of these symptoms are related to extant targets for treatment within the CHR syndrome as discussed in the introduction, for example improving ability to communicate around emotion, set boundaries, or improve social functioning. However, without directly addressing the traumatic etiology of these symptoms, treatment may be less effective.

Furthermore, the current literature examining trauma in the prodrome often focuses on the type and number of types of trauma experienced, with less attention paid to the intensity or chronicity of trauma (6). A body of work indicates a variety of other factors, including developmental timing, play a big role (38, 48–52). Some research includes subjective stress ratings which allows for examination of the perceived intensity of the trauma(s). However, little consideration has been made for the chronicity of traumas, in which repeated exposure may lead to complex trauma and the related complex PTSD (cPTSD) or DTD (53). Complex trauma can also result from multiple exposures to different traumas, which is especially relevant for the CHR population given the role of multiple trauma exposures in increased risk for developing a psychotic disorder. In fact, compared to PTSD, a diagnosis of cPTSD was found to be four times more common in a sample of individuals with psychosis (54). From a treatment perspective, this emphasizes the need for considerations beyond traditional PTSD symptoms.

## 5. Future directions

While a systematic review of the literature was outside the scope of our goals for this discussion piece, we endeavored to include perspectives from a broad number of disparate areas (including literature reviews and selection of recent as well as original landmark papers in domains including clinical high-risk, formal psychosis, and treatment for trauma). This approach was invaluable for informing our understanding of how trauma is currently conceptualized in the prodromal syndrome as well as highlighting unique treatment considerations for those with trauma histories, and promising future directions for interventions in this area. As this subfield continues to evolve and the body of available area-specific literature grows, systematic and qualitative review approaches will be necessary for the critical perspective provided by aggregating findings across studies.

In this perspective, we have highlighted that assessment of trauma exposure has historically been limited in granularity and often overlooked the critical aspect of timing of trauma within a developmental context. In addition, intensity and chronicity of traumas has received less attention than is merited considering the role these factors have in the development of trauma-related symptoms. With trauma exposure increasing risk for a considerable number of symptoms and increasing complexity of clinical presentations (24, 55), addressing these symptoms with trauma-focused treatment is of paramount importance. Additionally, consideration of the impacts of systemic and aggregate levels of trauma (e.g., crime exposure, ethnic density, poverty) have until recently been difficult to assess and rarely considered despite their influence in an individual’s level of risk for developing psychosis (38). These exposures and their timing within a developmental context must be integrated into assessment and treatment development for the CHR population, particularly given the differential impact on marginalized groups.

At the structural level, there are also considerable barriers to the access of care. These include but are not limited to difficulty navigating the mental health care system, difficulty getting connected to specialty care (if warranted), cost- or insurance-related barriers, documentation status related barriers, stigma, and lack of access to appropriate care (56–59). While these barriers are not limited to the treatment of psychosis-related symptoms, there is often additional stigma, misunderstanding of symptoms, and need for specialty care, which further exacerbate barriers (60). It is critical that, as in intervention for systems-level stressors, structural-level solutions are implemented to address structural-level barriers to accessing care to improve reach and clinical outcomes in this population.

Integrating comprehensive assessment for and treatment of trauma in the CHR population has the potential to greatly reduce disease burden by possibly improving treatment outcomes and reducing the probability of converting to a psychotic disorder. This initiative is only more important in the wake of the COVID-19 pandemic, which resulted in increased prevalence of exposure to trauma at a global level. While current trauma treatments for individuals with a CHR syndrome are limited, our understanding of broad trauma treatment and trauma treatments from the schizophrenia literature may serve as a foundation in tailoring intervention to the unique considerations of the CHR population.

## Data availability statement

The original contributions presented in this study are included in the article/supplementary material, further inquiries can be directed to the corresponding author.

## Author contributions

VZ wrote and edited the manuscript. TG and VM provided feedback and edited the manuscript. All authors contributed to the article and approved the submitted version.
